# Supplementary material for: Flexible brain dynamics underpins complex behaviours as observed in Parkinson’s disease
Source: Sci Rep. 2021 Feb 18;11:4051. doi: 10.1038/s41598-021-83425-4 (PMC7892831; doi:10.1038/s41598-021-83425-4)

**Flexible brain dynamics underpins complex behaviours as observed in Parkinson’s disease**

Pierpaolo Sorrentino^1,2,3, †,*^, Rosaria Rucco^4,3, †^, Fabio Baselice^1^, Rosa De Micco^5^, Alessandro Tessitore^5^, Arjan Hillebrand^6^, Laura Mandolesi^7^, Michael Breakspear^8^, Leonardo L. Gollo^2,9, ‡^, Giuseppe Sorrentino^3,4,10, ‡^

^†^ These authors contributed equally to the manuscript

^‡^ These authors contributed equally to the manuscript

1. Department of Engineering, University of Naples Parthenope, Centro Direzionale, Isola C4, 80143, Naples, Italy

2. QIMR Berghofer, 300 Herston Rd, Brisbane City, QLD 4006, Australia

3. Institute for Applied Science and Intelligent Systems, National Research Council, Via Campi Flegrei 34, Pozzuoli, Italy

4. Department of Motor Sciences and Wellness, University of Naples Parthenope, Via Ammiraglio Ferdinando Acton, 38, 80133, Naples, Italy

5. Department of Advanced Medical and Surgical Sciences, University of Campania “Luigi Vanvitelli”, via Luciano Armanni 5, 80138, Naples, Italy

6. Department of Clinical Neurophysiology and MEG Center, Amsterdam UMC, Vrije Universiteit
Amsterdam, Amsterdam Neuroscience, the Netherlands, De Boelelaan 1117, 1081HV Amsterdam, The Netherlands

7. Department of Humanistic Studies, University of Naples Federico II, via Porta di Massa 1, 80133 Naples, Italy

8. Priority Research Centre for Brain and Mind, The University of Newcastle, Medical Sciences, University Drive, Callaghan NSW 2308, Australia.

9. The Turner Institute for Brain and Mental Health, School of Psychological Sciences, and Monash Biomedical Imaging, Monash University, Victoria, Australia

10. Hermitage-Capodimonte Hospital, via Cupa delle Tozzole 2, Naples, Italy

Corresponding author: Dr. Pierpaolo Sorrentino. Email: pierpaolo.sorrentino@uniparthenope.it

**Supplementary Figures**

**Supplementary figure 1.** Differences in the size of functional repertoire in Parkinson patients (PD) and healthy controls (HC) with different binnings. On the top, no binning (binning = 1). On the bottom, binning =5.

**
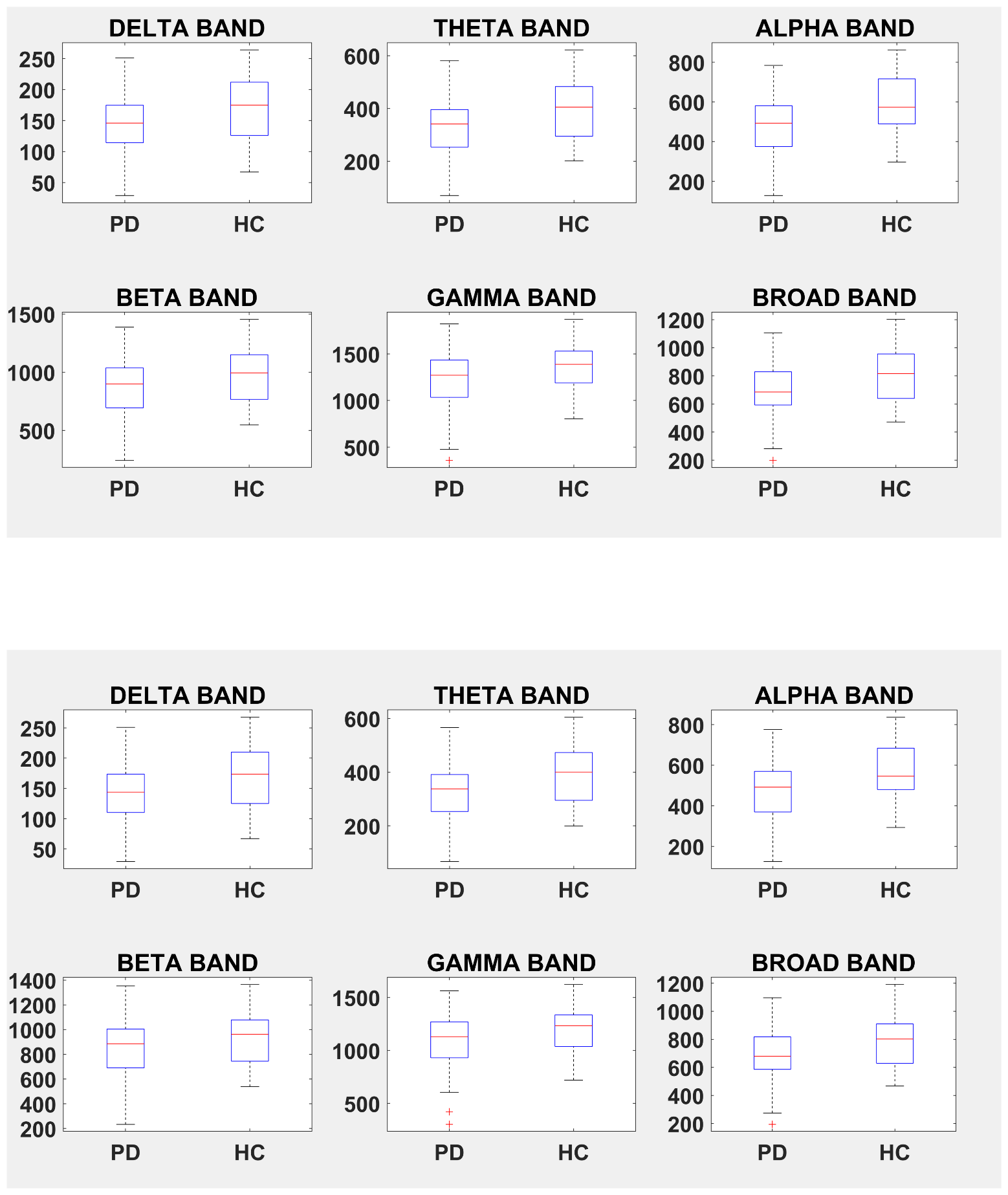
**

**Supplementary figure 2.** Differences in the size of the functional repertoire in Parkinson patients (PD) and healthy controls (HC) with different thresholds. On the top, threshold = 2.5. On the bottom, threshold = 3.5.


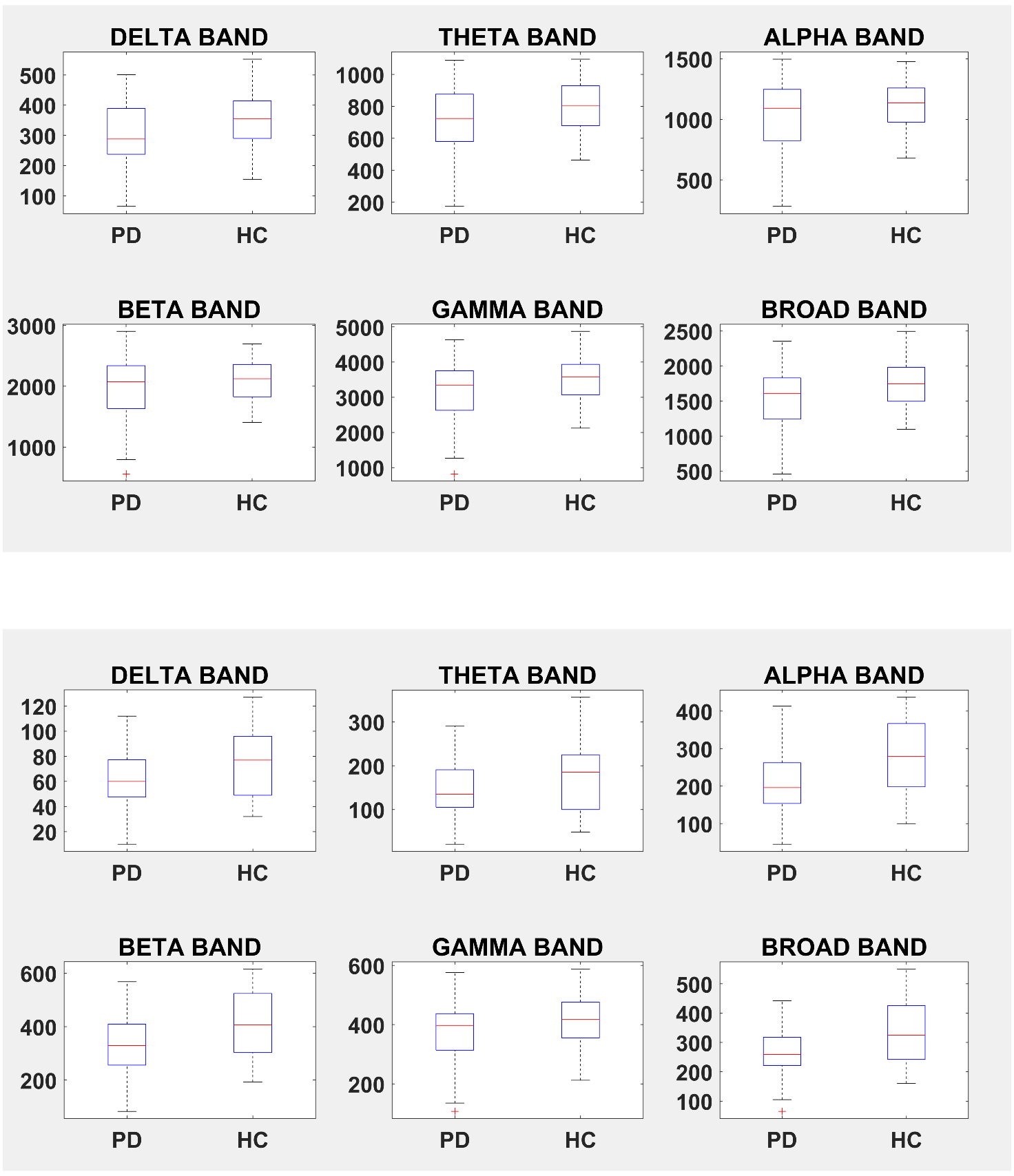


**Supplementary figure 3.** Differences in the size of the functional repertoire in Parkinson patients (PD) and healthy controls (HC), taking into account 90 brain areas (top) and 116 brain areas (bottom).

**
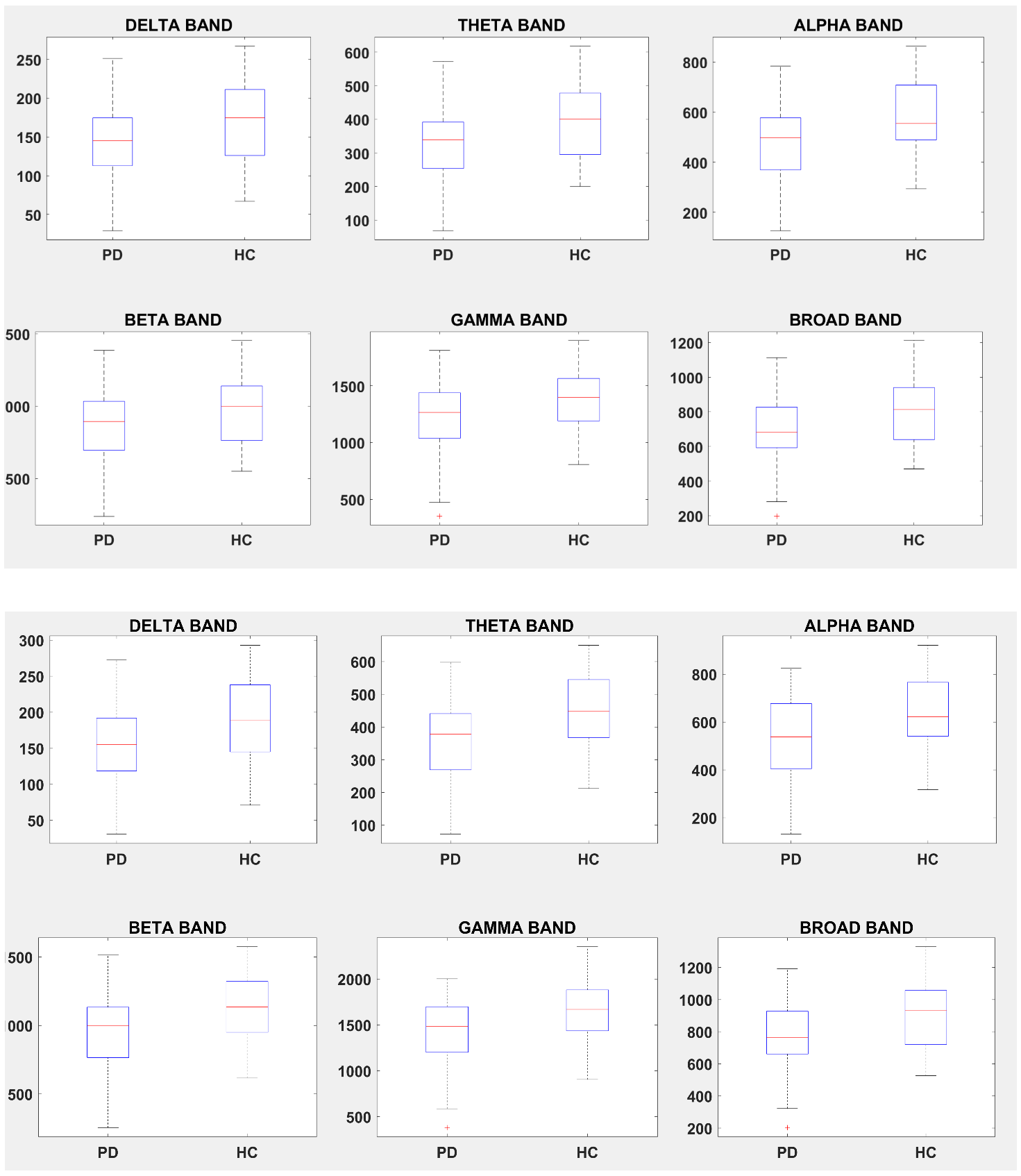
**

**Supplementary figure 4.** Differences in the size of the functional repertoire in Parkinson patients (PD) and healthy controls (HC), with the same length of data for each participant.


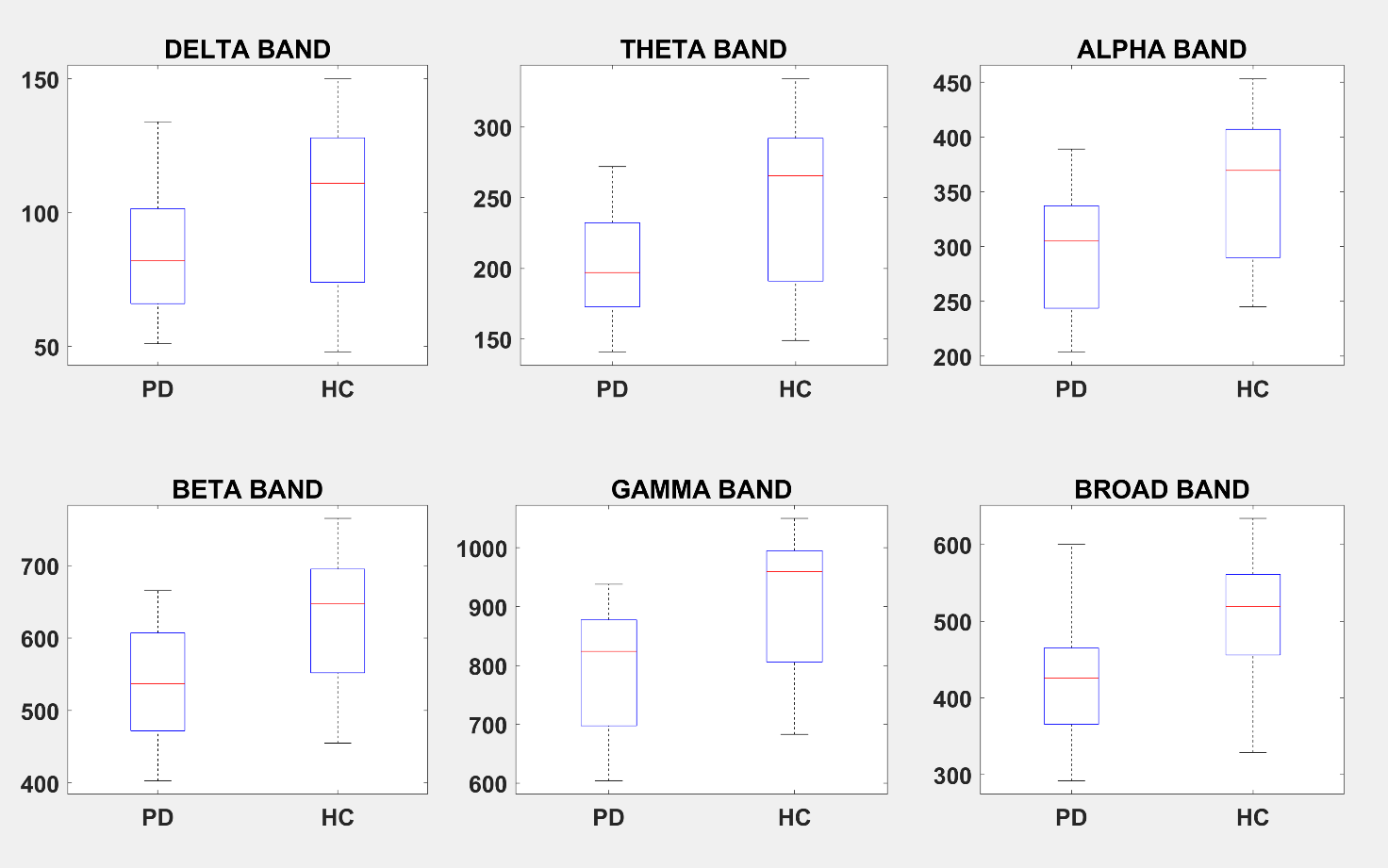

Supplement: Supplementary file 1 — Supplementary Figures. [file 41598_2021_83425_MOESM1_ESM.docx]
